# TyG-WHtR predicts incident type 2 diabetes mellitus in NAFLD: a 12-year prospective cohort study

**DOI:** 10.3389/fendo.2026.1805902

**Published:** 2026-05-01

**Authors:** Nan’nan Chen, Jiang Li, Gang Li, Faming Zhao

**Affiliations:** 1 Department of Pharmacy, Zibo Infectious Disease Hospital, Zibo, China; 2 Department of Cardiology, Affiliated Cardiovascular and Cerebrovascular Diseases Hospital of Yan’an University, Yan’an, China; 3 Department of Infectious Disease, Zibo Infectious Disease Hospital, Zibo, China

**Keywords:** Metabolic composite indices, nonalcoholic fatty liver disease, prospective cohort, triglyceride-glucose-waist-to-height ratio, type 2 diabetes mellitus

## Abstract

**Background:**

Metabolic composite indices effectively predict type 2 diabetes mellitus (T2DM) in the general population. However, whether these indices retain predictive value in patients with nonalcoholic fatty liver disease (NAFLD)—a population characterized by profound insulin resistance and metabolic heterogeneity—remains unclear. This study aimed to compare the predictive ability of twelve metabolic composite indices for the onset of T2DM in the NAFLD population.

**Methods:**

We conducted a secondary analysis of 2,370 NAFLD patients from a prospective Japanese cohort. Multivariate Cox proportional hazards models, subgroup analyses, and restricted cubic spline (RCS) regression were used to evaluate the associations between the twelve metabolic composite indices and incident T2DM. Predictive performance was assessed using receiver operating characteristic (ROC) and area under the curve (AUC) analyses, with optimal cut-offs determined using Youden’s index and validated by Kaplan–Meier curves.

**Results:**

In the fully adjusted model, all twelve indices were significantly associated with new-onset T2DM. The triglyceride–glucose–waist circumference (TyG-WC) index exhibited the greatest increase in the hazard ratio (HR) per 1-standard deviation (SD) [HR: 1.80, 95% confidence interval (CI): 1.56–2.07], followed by the triglyceride–glucose–waist–height ratio (TyG-WHtR) (HR: 1.77, 95% CI: 1.54–2.04) and the triglyceride–glucose–body–mass index (TyG-BMI) (HR: 1.70, 95% CI: 1.47–1.96). Conversely, the visceral adiposity index (VAI) showed the weakest association (HR per 1-SD increase: 1.29; 95% CI: 1.15–1.42). All associations were linear (*P* for nonlinearity >0.05 for all indices), and subgroup analyses revealed no significant interactions (*P* for interactions >0.05 for all). Notably, the TyG-WHtR exhibited the highest predictive accuracy (AUC = 0.680; optimal cut-off: 4.54), although its discriminative ability was not statistically superior to that of the TyG-WC index (*P* = 0.492). Its discriminatory ability remained stable throughout the follow-up period, and AUC values ranged from 0.634 to 0.719.

**Conclusions:**

All the assessed metabolic composite indices are linearly associated with incident T2DM in NAFLD patients. The TyG-WHtR may serve as a practical tool for T2DM risk stratification in patients with NAFLD, given its moderate yet temporally consistent discriminatory ability and easily obtainable components.

## Introduction

With considerable changes in lifestyle and urbanization, the global burden of metabolic diseases has continued to increase. Nonalcoholic fatty liver disease (NAFLD) has become the leading cause of chronic liver disease worldwide. According to a report by Zobair M. Younossi et al., the global incidence of NAFLD increased from 25.16% from 1990–2006 to 34.59% from 2016–2019 (1). By 2040, approximately half of the global population is projected to be affected by NAFLD (2). Beyond its high prevalence, NAFLD contributes substantially to liver-related morbidity and mortality worldwide. Studies have shown that individuals with NAFLD are more prone to experience symptoms such as fatigue, depression, and impaired physical functioning than are healthy controls (3). Moreover, all-cause mortality among NAFLD patients is 93% higher than that in the general population (4). The economic burden associated with NAFLD is also considerable, with estimated annual direct health care costs of approximately €35 billion in Europe and $ 101 billion in the United States (5). These figures underscore the profound impact of NAFLD on global public health.

More than a decade ago, Byrne et al. (6) emphasized that NAFLD is a multisystem disorder that not only increases the risk of liver-related complications—including nonalcoholic steatohepatitis (NASH), hepatic fibrosis, cirrhosis, and hepatocellular carcinoma—but also heightens susceptibility to type 2 diabetes mellitus (T2DM) (5, 7, 8). Accumulating evidence has since confirmed a strong association between NAFLD and T2DM (9, 10). Up to 75% of patients with T2DM have concomitant NAFLD, while the prevalence of T2DM among adults with NAFLD ranges from 10% to 18%, which markedly exceeds that in the general population (11–13). Some theories even propose that NAFLD may represent a prediabetic state (14). Compared with NAFLD, T2DM is associated with more frequent and severe clinical consequences, including diabetic retinopathy (DR, a cause of blindness) and diabetic foot ulcers, which are the most common causes of nontraumatic lower-limb amputations (15). Furthermore, T2DM significantly increases the risks of both all-cause and cardiovascular mortality (16, 17). Importantly, early-stage NAFLD can often be reversed through weight loss (18), whereas once T2DM is established, it is generally considered irreversible. Therefore, timely identification of individuals with NAFLD who are at high risk for the development of T2DM—and implementation of proactive interventions—has critical clinical significance.

Insulin resistance (IR) plays an essential role in the pathogenesis and progression of both T2DM and NAFLD. Although the hyperinsulinaemic-euglycaemic clamp remains the gold standard for assessing IR, its invasiveness, high cost, and operational complexity limit its utility in routine clinical practice and in large-scale studies. In recent years, several metabolic composite indices have emerged as convenient, noninvasive, and clinically feasible surrogate markers of IR. These indices include the triglyceride-glucose (TyG) index, triglyceride-glucose-waist-circumference (TyG-WC), triglyceride-glucose-body-mass index (TyG-BMI), triglyceride-glucose-body-roundness index (TyG-BRI), cholesterol-high-density-lipoprotein-glucose (CHG) index, lipid accumulation product (LAP), visceral adiposity index (VAI) and so on. Recently, Dou et al. performed a comprehensive evaluation of multiple metabolic composite indices in a large, general Japanese cohort (19). While their findings provide valuable data, whether these results can be extrapolated to NAFLD patients—who experience profound metabolic abnormalities and insulin resistance—remains uncertain. Therefore, this study aimed to systematically compare and validate the predictive ability of twelve metabolic composite indices for incident T2DM in the population with NAFLD.

## Methods

### Data source and study population

This study conducted a secondary analysis of data obtained from the Dryad Digital Repository (https://datadryad.org), a publicly accessible research data archive. The original dataset was derived from a prospective cohort study, NAFLD in the Gifu area longitudinal analysis (NAGALA), by Okamura et al. (19). The database includes demographic, clinical, and biochemical variables such as age, sex, body weight, systolic and diastolic blood pressure, waist circumference (WC), obesity phenotype, fasting plasma glucose (FPG) level, body mass index (BMI), the levels of haemoglobin A1c (HbA1c), triglyceride (TG), total cholesterol (TC), high-density lipoprotein cholesterol (HDL-c), alanine aminotransferase (ALT), aspartate aminotransferase (AST), and gamma-glutamyl transferase (GGT) as well as alcohol consumption, smoking status, exercise habits, fatty liver status, follow-up duration, and incident diabetes.

**Figure 1:**
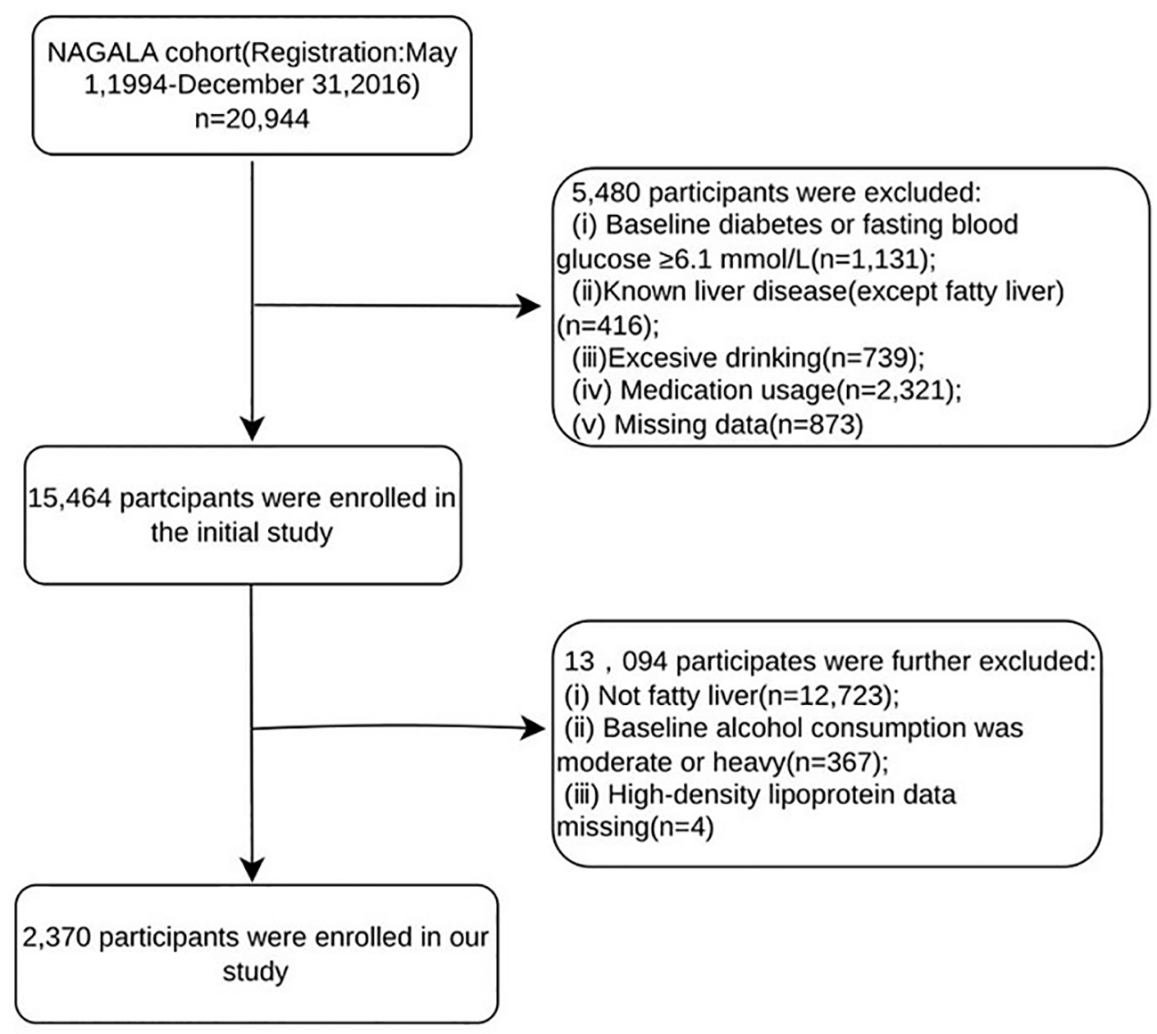
Participants screening flowchart.

The parent study enrolled individuals who underwent health examinations at Murakami Memorial Hospital (Gifu, Japan) between 2004 and 2015. After the exclusion criteria described by Okamura et al. (baseline diabetes or impaired fasting glucose, known liver disease other than fatty liver, excessive alcohol intake, medication use, and missing data) were applied, 15,464 participants were included in the final cohort. For the present analysis, we further excluded 13,094 individuals who did not have fatty liver (n=12,723), reported moderate or heavy alcohol consumption at baseline (n=367), or had missing HDL-c data (n=4). Thus, the final study population comprised 2,370 participants with NAFLD but no prior diagnosis of T2DM at baseline, as shown in **Figure 1**.

The original study protocol was approved by the Institutional Review Board of Murakami Memorial Hospital, and all participants provided written informed consent (19). As this is a secondary analysis of anonymized data, the requirement for additional ethical approval and informed consent was waived by the Ethics Committee of Zibo Infectious Disease Hospital. This study was conducted in accordance with the ethical standards of the 1964 Declaration of Helsinki and its later amendments, as well as the international ethical guidelines for health-related research involving human subjects (e.g., CIOMS International Ethical Guidelines).

### Definitions of exposures, outcomes, and covariates

We examined twelve metabolic composite indices associated with T2DM in NAFLD patients, the relevant formulas for which are provided in **Supplementary Table 1**. The primary outcome was incident T2DM, defined as an FPG ≥7.0 mmol/L, an HbA1c ≥6.5%, or a self-reported physician diagnosis during follow-up.

Fatty liver was diagnosed via abdominal ultrasonography performed by trained technicians. NAFLD was defined as the presence of hepatic steatosis in the absence of other chronic liver diseases, including hepatitis B or C infection and a consumption of <140 g/week of alcohol.

Covariates considered for adjustment included age, sex, ALT level, AST level, GGT level, smoking status, hypertension, and exercise habits. Hypertension was defined as a systolic blood pressure ≥140 mmHg and/or a diastolic blood pressure ≥90 mmHg. Patients were categorized as never, former, or current smokers according to their smoking status. Regular exercise was defined as engagement in sports activities more than once per week. Twelve metabolic composite indicators were included in this study.

### Statistical analysis

Continuous variables were assessed for normality using visual inspection of distributions. Normally distributed data are presented as the mean ± standard deviation (SD), while nonnormally distributed data are presented as the median (interquartile range, IQR). Categorical variables are summarized as frequencies and percentages.

Group comparisons were performed using Student’s t test (normal continuous variables), the Mann–Whitney U test (nonnormal continuous variables), or the chi-square test (categorical variables). Multivariate Cox proportional hazards regression models were used to estimate hazard ratios (HRs) and 95% confidence intervals (CIs) for the association between each metabolic composite index and incident T2DM. The proportional hazards assumption for the Cox regression models was evaluated using Schoenfeld residuals. This assumption was satisfied for all included variables (see **Supplementary Table 2A**). The unadjusted model was followed by Model 1 (adjusted for age and sex) and Model 2 (adjusted for age, sex, ALT, AST, GGT, smoking status, hypertension, and exercise habits). To avoid any potential overadjustment bias, no glycaemic parameters (including either FPG or HbA1c) were included. Due to the high correlation among components of the composite indices, separate Cox proportional hazards models were constructed for each index to evaluate their independent associations with T2DM, and thus each index was not included in a single multivariable model. Multicollinearity among covariates was assessed by calculating variance inflation factors (VIFs). No evidence of significant multicollinearity was observed, as all the VIF values were well below the threshold of 5. Detailed VIF values for each variable in every model are provided in **Supplementary Table 2B**. For all indicators, in addition to per-unit analysis, the HRs per-SD increase were also calculated to facilitate clinical interpretation and comparison. Data completeness was verified prior to analysis. No values for any of the study variables were missing in the included patient cohort; thus, the analysis was conducted on the complete dataset without the need for imputation methods.

To examine consistency across subpopulations, stratified analyses were conducted by age (<45 vs. ≥45 years), sex, smoking status, exercise habits, and hypertension status. Interaction effects were tested using likelihood ratio tests. Dose–response relationships were explored using restricted cubic splines (RCSs) with four knots.

The predictive performance of the metabolic composite indices for incident T2DM was assessed using receiver operating characteristic (ROC) curve analysis. The discriminative ability of the indices was compared by calculating the area under the curve (AUC), with the statistical significance of differences evaluated via the DeLong test. For the index with the highest AUC, the optimal cut-off value was determined on the basis of the maximum Youden’s index. Participants were then stratified into low- and high-risk groups according to this cut-off, and the cumulative incidence of T2DM between the groups was visualized using Kaplan–Meier curves, with differences compared by the log-rank test. Additionally, time-dependent ROC curve analysis was conducted to evaluate the longitudinal predictive accuracy of this index over the 1- to 12-year follow-up period.

All analyses were performed using R software version 4.2.2 (R Foundation for Statistical Computing) and Empower Stats (X&Y Solutions, Inc.). A two-tailed *P* value <0.05 was considered to indicate statistical significance.

## Results

### Baseline characteristics of the study population

In all, 2,370 participants with a diagnosis of NAFLD (mean age 44.65 ± 8.31 years) were included in this study. The follow-up duration ranged from 0.5 to 12.96 years, with a mean follow-up duration of 5.97 years. During follow-up, 198 individuals (8.35%) developed T2DM. As shown in **Table 1**, compared with those who did not develop T2DM, participants who progressed to T2DM presented significantly higher baseline levels of TC, TG, FPG, HbA1c, ALT, AST, and GGT as well as higher BMI, body weight, and WC and were more likely to be current smokers (all *P* < 0.05). Furthermore, all twelve metabolic composite indices assessed in this study were significantly elevated in the T2DM group. In contrast, HDL-c levels were significantly lower in individuals who developed T2DM.

**Table 1:** Patients’ demographics and baseline characteristics.

Variables	Overall (n=2370)	Non-T2DM group(n=2172)	T2DM-group (n=198)	*P*-value
Age (years)	44.65 ± 8.31	44.55 ± 8.35	45.79 ± 7.74	0.045^1^
Follow-up time(years)	5.97± 3.78	5.98± 3.83	5.83± 3.20	0.601
ALT(IU/L)	27.00 (20.00-39.00)	26.00 (20.00-39.00)	31.00 (23.00-45.00)	<0.001^2^
AST(IU/L)	20.00 (17.00-26.00)	20.00 (16.75-25.00)	21.00 (17.00-27.75)	0.008^2^
GGT(IU/L)	22.00 (16.00-32.00)	22.00 (16.00-32.00)	25.00 (18.00-37.00)	<0.001^2^
TC (mg/dL)	210.56 ± 33.47	210.09 ± 33.26	215.69 ± 35.31	0.024^1^
HbA1-c (%)	5.31 ± 0.33	5.28 ± 0.32	5.56 ± 0.34	<0.001^1^
TG (mg/dL)	109.00 (77.00-158.00)	108.00 (76.00-153.00)	133.00 (90.00-194.00)	<0.001^2^
FPG (mg/dL)	97.08 ± 6.56	96.67 ± 6.47	101.65 ± 5.85	<0.001^1^
HDL-c (mg/dL)	45.76 ± 11.07	46.06 ± 11.16	42.45 ± 9.33	<0.001^1^
BMI (kg/m^2^)	25.52 ± 3.16	25.41 ± 3.10	26.75 ± 3.57	<0.001^1^
Weight (kg)	72.11 ± 11.44	71.80 ± 11.32	75.47 ± 12.16	<0.001^1^
WC (cm)	85.94 ± 7.86	85.59 ± 7.69	89.81 ± 8.68	<0.001^1^
AIP	0.03 ± 0.29	0.02 ± 0.29	0.13 ± 0.31	<0.001^1^
CHG	5.42 ± 0.28	5.41 ± 0.28	5.56 ± 0.27	<0.001^1^
CMI	0.70 ± 0.54	0.68 ± 0.52	0.93 ± 0.68	<0.001^1^
LAP	32.52 ± 23.12	31.41 ± 22.25	44.69 ± 28.49	<0.001^1^
METS-IR	38.89 ± 6.27	38.59 ± 6.12	42.20 ± 6.98	<0.001^1^
TyG	8.58 ± 0.55	8.56 ± 0.54	8.79 ± 0.58	<0.001^1^
TYG-BRI	31.26 ± 8.38	30.81 ± 8.05	36.16± 10.19	<0.001^1^
TyG-BMI	219.17 ± 32.29	217.70 ± 31.54	235.32 ± 35.89	<0.001^1^
TyG-WC	737.93 ± 89.02	733.19 ± 86.95	789.92 ± 94.91	<0.001^1^
TyG-WHtR	4.40 ± 0.51	4.37 ± 0.50	4.71 ± 0.56	<0.001^1^
TyG-WWI	87.11 ± 7.27	86.74 ± 7.12	91.17 ± 7.61	<0.001^1^
VAI	1.82 ± 1.37	1.77 ± 1.33	2.35 ± 1.64	<0.001^1^
Gender (n, %)				0.844^3^
Male	1892 (79.83%)	1735 (79.88%)	157 (79.29%)	
Female	478 (20.17%)	437 (20.12%)	41 (20.71%)	
Habit of exercise (n, %)				0.046^3^
No	2016 (85.06%)	1838 (84.62%)	178 (89.90%)	
Yes	354 (14.94%)	334 (15.38%)	20 (10.10%)	
Smoking (n, %)				<0.001^3^
Never	1149 (48.48%)	1072 (49.36%)	77 (38.89%)	
Former	582 (24.56%)	538 (24.77%)	44 (22.22%)	
Current	639 (26.96%)	562 (25.87%)	77 (38.89%)	
HBP (n, %)				0.277^3^
No	2036 (85.91%)	1871 (86.14%)	165 (83.33%)	
Yes	334 (14.09%)	301 (13.86%)	33 (16.67%)	

ALT, alanine aminotransferase; AST, aspartate transaminase; GGT, gamma-glutamyl transferase; TC, total cholesterol; HbA1-c, hemoglobin a1c; TG, triglyceride; FPG, fasting plasma glucose; HDL-c, high-density lipoprotein cholesterol; BMI, body mass index; WC, Waist circumference; WHtR, waist-to-height ratio; AIP, atherogenic index of plasma; BRI, body roundness index; CHG, cholesterol, high density lipoprotein, and glucose index; CMI, cardiometabolic index; LAP, lipid accumulation product; METS-IR, metabolic score for insulin resistance; TyG, triglyceride-glucose index; WWI, weight-adjusted-waist index; VAI, visceral adiposity index; HBP, hypertension.

^1^Mean ± SD; One-way ANOVA.

^2^Median (Q1-Q3); Kruskal-Wallis H test.

^3^n (%); Pearson’s Chi-squared test.

### Associations between metabolic composite indices and incident T2DM

**Table 2** presents the associations of the twelve metabolic composite indices with incident T2DM among NAFLD patients. In the multivariable Cox regression analysis, all indices were significantly associated with new-onset T2DM in both the unadjusted and adjusted models (all *P* < 0.05), and these associations persisted after full adjustment for potential confounders (Model 2). When assessed per 1-SD increase, the TyG-related indices exhibited large effect sizes. The TyG-WC index showed the highest increase in the HR per 1-SD increase [HR: 1.80, 95% CI: 1.56–2.07], followed closely by the triglyceride-glucose-waist-to-height ratio (TyG-WHtR) (HR: 1.77, 95% CI: 1.54–2.04) and the TyG-BMI (HR: 1.70, 95% CI: 1.47–1.96). In contrast, the VAI demonstrated the weakest association (HR per 1-SD: 1.29; 95% CI: 1.15–1.42). Other indices, including metabolic score for insulin resistance (METS-IR), LAP, and atherogenic index of plasma (AIP), showed moderate effect sizes, and per-SD HRs ranged from 1.34 to 1.66 (all *P*<0.01). When the indices were modelled as continuous variables per 1-unit increase, the magnitude of the HRs varied substantially because of differences in their measurement scales. For instance, the TyG-WHtR exhibited a relatively large unit HR (HR: 3.06; 95% CI: 2.32–4.04), which indicates that each 1-unit increase in this index was associated with a 3.06-fold increased risk of T2DM. This large effect per unit reflects the relatively narrow distribution of the TyG-WHtR in our cohort, which suggests that even minor absolute changes in this index carry substantial prognostic information. Conversely, indices with wider scales, such as the TyG-WC index (HR per unit: 1.01, 95% CI: 1.01–1.01), showed modest per-unit HRs despite their strong per-SD associations; therefore, a 1-unit increase in TyG-WC corresponded to only a 1% increase in T2DM risk.

**Table 2:** Multivariable Cox proportional hazards regression analysis.

Variables	Unadjusted model		Model 1		Model 2	
	HR (95%CI)	*P* value	HR (95%CI)	*P* value	HR (95%CI)	*P* value
TyG						
^1^Continuous	2.03 (1.57-2.63)	<0.001	2.21 (1.69-2.88)	<0.001	1.98 (1.50-2.62)	<0.001
^2^Continuous	1.48 (1.28-1.70)	<0.001	1.54 (1.33-1.79)	<0.001	1.46 (1.25-1.70)	<0.001
Categorized						
Low [6.56,8.34)	Reference		Reference		Reference	
Middle [8.34,8.81)	1.31 (0.87-1.97)	0.20	1.36 (0.90-2.05)	0.14	1.28 (0.85-1.93)	0.24
High [8.81-10.47]	2.46 (1.71-3.54)	<0.001	2.71 (1.87-3.95)	<0.001	2.27 (1.55-3.33)	<0.001
P for trend		<0.001		<0.001		<0.001
TyG-BRI						
^1^Continuous	1.06 (1.05-1.08)	<0.001	1.07 (1.05-1.08)	<0.001	1.06 (1.04-1.08)	<0.001
^2^Continuous	1.68 (1.51-1.87)	<0.001	1.71 (1.53-1.91)	<0.001	1.62 (1.44-1.84)	<0.001
Categorized						
Low [10.84,27.12)	Reference		Reference		Reference	
Middle [27.12,33.29)	1.94 (1.26-3.00)	0.003	1.86 (1.20-2.88)	0.005	1.75 (1.13-2.71)	0.012
High [33.29,86.87]	3.77 (2.52-5.63)	<0.001	3.62 (2.42-5.42)	<0.001	3.04 (2.01-4.61)	<0.001
P for trend		<0.001		<0.001		<0.001
TyG-BMI						
^1^Continuous	1.02 (1.01-1.02)	<0.001	1.02 (1.01-1.02)	<0.001	1.02 (1.01-1.02)	<0.001
^2^Continuous	1.64 (1.45-1.84)	<0.001	1.78 (1.57-2.03)	<0.001	1.70 (1.47-1.96)	<0.001
Categorized						
Low [131.17,203.68)	Reference		Reference		Reference	
Middle[203.68-230.14)	1.59 (1.05-2.42)	0.03	1.65 (1.08-2.52)	0.02	1.50 (0.98-2.30)	0.06
High [230.14,421.35]	3.31 (2.26-4.86)	<0.001	3.76 (2.55-5.53)		3.14 (2.10-4.72)	<0.001
P for trend		<0.001		<0.001		<0.001
TyG-WC						
^1^Continuous	1.01 (1.01-1.01)	<0.001	1.01 (1.01-1.01)	<0.001	1.01 (1.01-1.01)	<0.001
^2^Continuous	1.76 (1.56-2.02)	<0.001	1.91 (1.67-2.18)	<0.001	1.80 (1.56-2.07)	<0.001
Categorized						
Low [440.11,697.66)	Reference		Reference		Reference	
Middle [697.66,770.64)	1.39 (0.90-2.15)	0.11	1.64 (1.04-2.57)	0.03	1.48 (0.94-2.33)	0.09
High [770.64,1097.18]	3.36 (2.29-4.93)	<0.001	4.12 (2.75-6.17)	<0.001	3.44 (2.26-5.24)	<0.001
P for trend		<0.001		<0.001		<0.001
TyG-WHtR						
^1^Continuous	3.43 (2.66-4.41)	<0.001	3.45 (2.67-4.46)	<0.001	3.06 (2.32-4.04)	<0.001
^2^Continuous	1.88 (1.65-2.14)	<0.001	1.88 (1.65-2.15)	<0.001	1.77 (1.54-2.04)	<0.001
Categorized						
Low [2.62,4.17)	Reference		Reference		Reference	
Middle [4.17,4.58)	1.44 (0.92-2.25)	0.11	1.42 (0.91-2.23)	0.12	1.32 (0.84-2.07)	0.23
High [4.58,6.59]	3.97 (2.68-5.87)	<0.001	3.95 (2.67-5.84)	<0.001	3.32 (2.21-5.00)	<0.001
P for trend		<0.001		<0.001		<0.001
TyG-WWI						
^1^Continuous	1.09 (1.07-1.11)	<0.001	1.08 (1.06-1.10)	<0.001	1.07 (1.05-1.09)	<0.001
^2^Continuous	1.83 (1.59-2.01)	<0.001	1.78 (1.55-2.06)	<0.001	1.65 (1.42-1.92)	<0.001
Categorized						
Low [57.74,83.77)	Reference		Reference		Reference	
Middle [83.77,90.20)	1.51 (0.99-2.31)	0.05	1.51 (0.98-2.31)	0.06	1.36 (0.88-2.08)	0.16
High [90.20,115.75]	3.11 (2.13-4.56)	<0.001	3.00 (2.04-4.43)	<0.001	2.47 (1.66-3.68)	<0.001
P for trend		<0.001		<0.001		<0.001
AIP						
^1^Continuous	2.88 (1.77-4.70)	<0.001	3.46 (2.09-5.72)	<0.001	2.70 (1.61-4.54)	0.002
^2^Continuous	1.37 (1.18-1.59)	<0.001	1.44 (1.24-1.67)	<0.001	1.34 (1.15-1.56)	0.002
Categorized						
Low [-0.93, -0.097)	Reference		Reference		Reference	
Middle [-0.097,0.15)	1.42 (0.96-2.11)	0.08	1.52 (1.02-2.26)	0.04	1.45 (0.97-2.16)	0.07
High [0.15,1.07]	2.06 (1.43-2.97)	<0.001	2.33 (1.60-3.41)	<0.001	1.94 (1.31-2.86)	<0.001
P for trend		<0.001		<0.001		<0.001
CHG						
^1^Continuous	5.83 (3.44-9.89)	<0.001	7.00 (4.07-12.04)	<0.001	5.26 (3.02-9.18)	<0.001
^2^Continuous	1.64 (1.41-1.90)	<0.001	1.72 (1.48-2.00)	<0.001	1.59 (1.36-1.86)	<0.001
Categorized						
Low [4.41,5.31)	Reference		Reference		Reference	
Middle [5.31,5.54)	2.12 (1.38-3.26)	<0.001	2.29 (1.48-3.54)	<0.001	2.18 (1.41-3.38)	<0.001
High [5.54,6.34]	2.94 (1.95-4.42)	<0.001	3.32 (2.17-5.06)	<0.001	2.87 (1.87-4.39)	<0.001
P for trend		<0.001		<0.001		<0.001
CMI						
^1^Continuous	1.73 (1.45-2.08)	<0.001	1.80 (1.50-2.16)	<0.001	1.57 (1.27-1.93)	<0.001
^2^Continuous	1.35 (1.22-1.48)	<0.001	1.37 (1.25-1.51)	<0.001	1.31 (1.18-1.46)	<0.001
Categorized						
Low [0.053,0.41)	Reference		Reference		Reference	
Middle [0.41,0.74)	1.33 (0.89-2.00)	0.16	1.43 (0.96-2.15)	0.08	1.30 (0.86-1.95)	0.214
High [0.74,6.17]	2.22 (1.54-3.20)	<0.001	2.49 (1.71-3.64)	<0.001	2.02 (1.37-2.98)	<0.001
P for trend		<0.001		<0.001		<0.001
LAP						
^1^Continuous	1.02 (1.01-1.02)	<0.001	1.02 (1.01-1.02)	<0.001	1.02 (1.01-1.02)	<0.001
^2^Continuous	1.49 (1.35-1.64)	<0.001	1.52 (1.38-1.68)	<0.001	1.44 (1.30-1.61)	<0.001
Categorized						
Low [0.34,19.72)	Reference		Reference		Reference	
Middle [19.72,35.54)	1.42 (0.92-2.20)	0.11	1.42 (0.92-2.19)	0.12	1.28 (0.82-1.98)	0.27
High [35.54,212.52]	3.32 (2.26-4.87)	<0.001	3.51 (2.39-5.17)	<0.001	2.92 (1.96-4.36)	<0.001
P for trend		<0.001		<0.001		<0.001
METS-IR						
^1^Continuous	1.08 (1.06-1.10)	<0.001	1.10 (1.07-1.12)	<0.001	1.08 (1.06-1.11)	<0.001
^2^Continuous	1.63 (1.44-1.84)	<0.001	1.78 (1.56-2.02)	<0.001	1.66 (1.44-1.91)	<0.001
Categorized						
Low [21.61,35.87)	Reference		Reference		Reference	
Middle [35.87,41.08)	1.16 (0.77-1.76)	0.48	1.25 (0.83-1.91)	0.29	1.13 (0.74-1.73)	0.57
High [41.08,73.16]	2.70 (1.87-3.88)	<0.001	3.10 (2.14-4.50)	<0.001	2.54 (1.73-3.74)	<0.001
P for trend		<0.001		<0.001		<0.001
VAI						
^1^Continuous	1.23 (1.14-1.32)	<0.001	1.23 (1.14-1.32)	<0.001	1.20 (1.11-1.29)	<0.001
^2^Continuous	1.32 (1.20-1.45)	<0.001	1.32 (1.20-1.46)	<0.001	1.29 (1.15-1.42)	<0.001
Categorized						
Low [0.17,1.11)	Reference		Reference		Reference	
Middle [1.11,1.92)	1.40 (0.94-2.09)	0.10	1.44 (0.96-2.15)	0.08	1.33 (0.88-1.99)	0.17
High [1.92,15.14]	2.21 (1.53-3.19)	<0.001	2.24 (1.55-3.24)	<0.001	1.87 (1.29-2.72)	0.001
P for trend		<0.001		<0.001		<0.001

Model1 adjust for sex, age.

Model2 adjust for sex, age, ALT, AST, Habit of exercise, GGT, Smoking status, HBP. (HbA1c was excluded to avoid overadjustment bias; see **Supplementary Table 2C** for results including the variable).

^1^HR per 1-unit increase.

^2^HR per 1-SD increase.

ALT, alanine aminotransferase; AST, aspartate transaminase; GGT, gamma-glutamyl transferase; HbA1-c, hemoglobin a1c; BMI, body mass index; WC, Waist circumference; WHtR, waist-to-height ratio; AIP, atherogenic index of plasma; BRI, body roundness index; CHG, cholesterol, high density lipoprotein, and glucose index; CMI, cardiometabolic index; LAP, lipid accumulation product; METS-IR, metabolic score for insulin resistance; TyG, triglyceride-glucose index; WWI, weight-adjusted-waist index; VAI, visceral adiposity index; HBP, hypertension.

After each index was categorized into tertiles, a clear dose–response relationship was observed. Compared with participants in the lowest tertile, participants in the highest tertile of each index had a significantly elevated risk of T2DM in the fully adjusted model, and significant linear trends were observed across all indices (*P* for all trends <0.05). For example, individuals in the highest tertile of TyG-WC had a 3.44-fold increased risk (HR: 3.44; 95% CI: 2.26–5.24), whereas those in the highest tertile of the VAI had a 1.87-fold increased risk (HR: 1.87; 95% CI: 1.29–2.72).

### Subgroup analyses

To assess the robustness of the primary findings, subgroup analyses were performed on the basis of age (<45 vs. ≥45 years), sex, smoking status, exercise habits, and hypertension status (**Supplementary Figure 1**). No significant interaction effects were observed across these subgroups (*P* for all interactions > 0.05), which indicates that the associations between metabolic composite indices and T2DM risk were consistent irrespective of these baseline characteristics.

### Dose–response relationships

RCS analyses were conducted to explore the shape of the dose–response relationship between each metabolic composite index and T2DM risk (**Supplementary Figure 2**). All indices exhibited an approximately linear association with T2DM incidence (*P* for all nonlinearity > 0.05).

### Predictive performance of metabolic composite indices for incident T2DM

**Figure 2:**
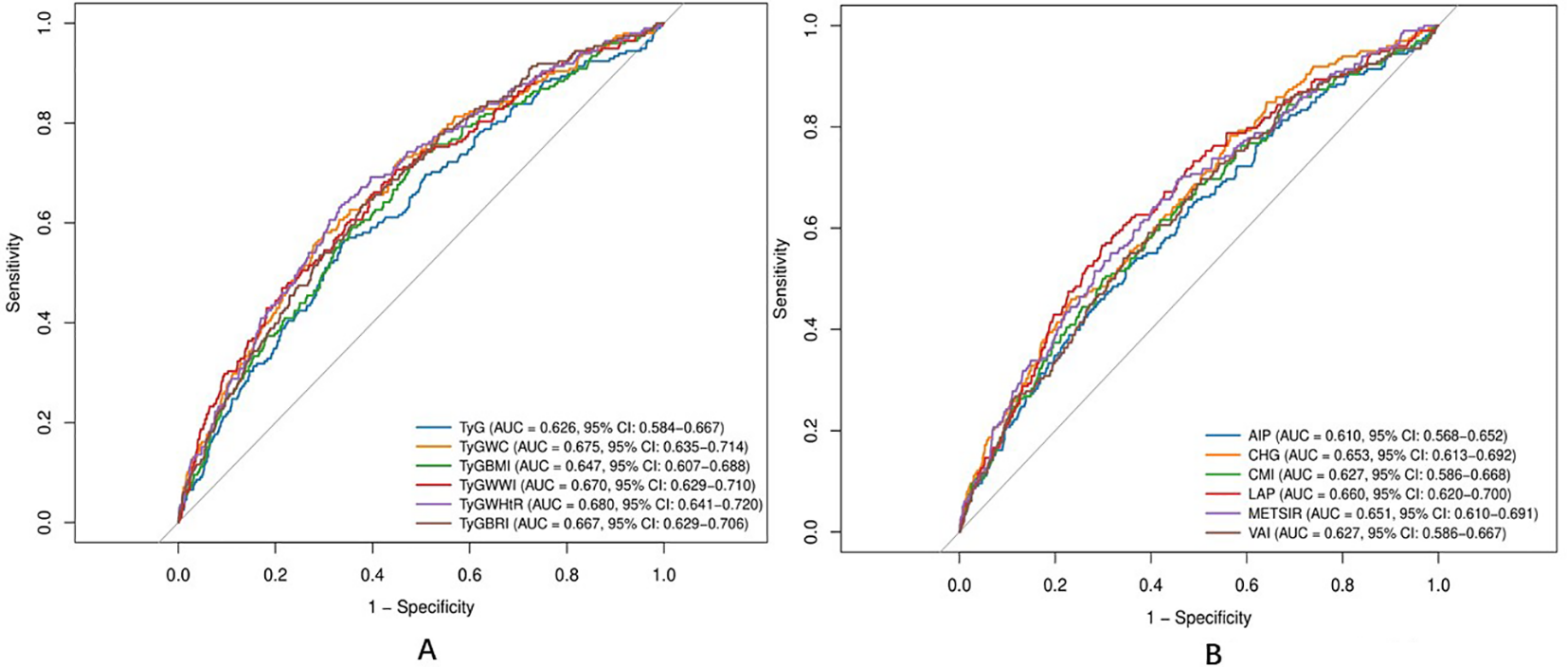
Receiver operating characteristics (ROC) analysis of predictive value for twelve metabolic composite indicators. **(A)** Triglyceride-glucose related indices. **(B)** Other indices.

**Figure 3:**
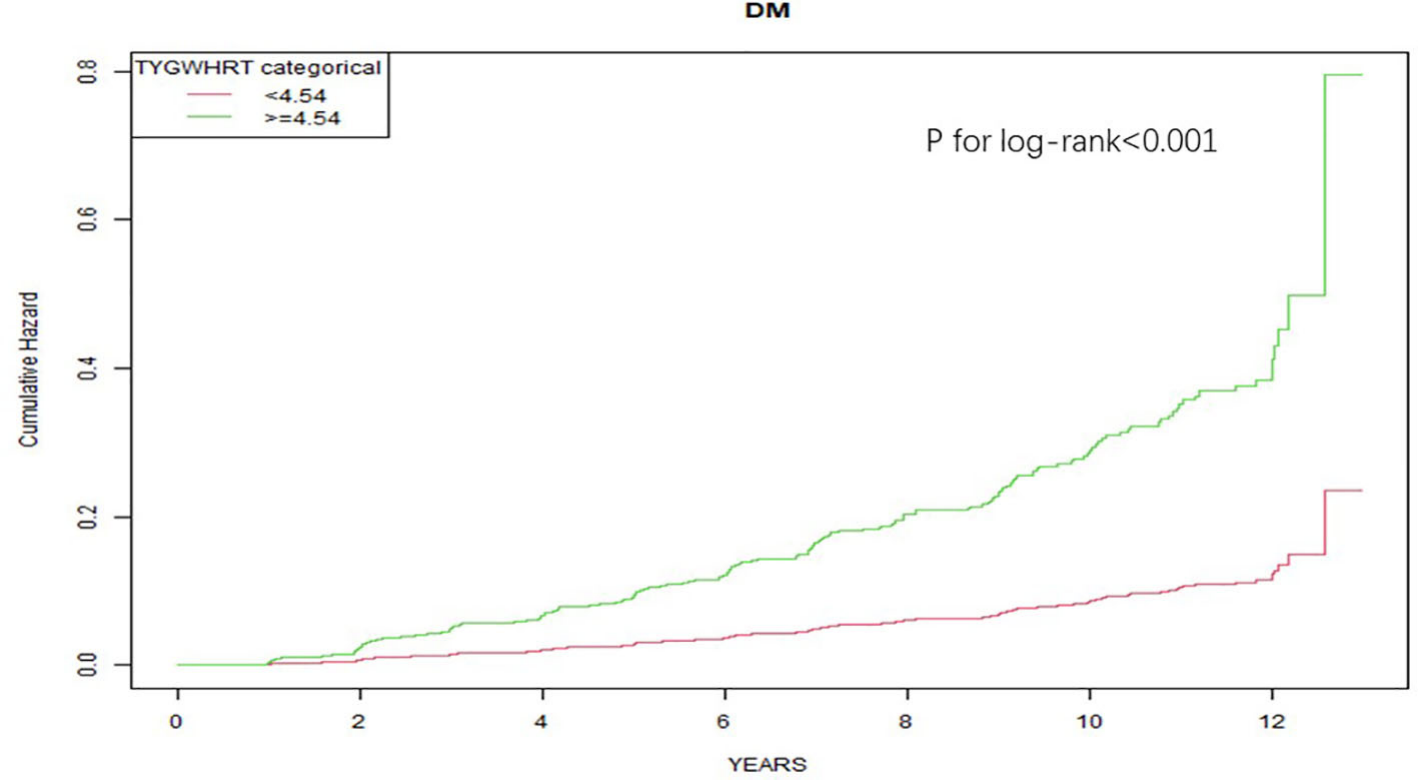
Kaplan-Meier curve for cumulating incidence of T2DM in NAFLD patients. Participants were satisfied into low-risk ( < 4.54) and high-risk ( ≥ 4.54) groups based on the TyG-WHtR cut-off value 4.54.

The ability of each metabolic composite index to predict incident T2DM was assessed using ROC analysis (**Figure 2**). The AUC of the TyG-WHtR was 0.680 (95%CI: 0.641–0.720) greater than the AUC values of the other indices, including TyG-WC (AUC = 0.675). However, pairwise comparisons using DeLong’s test revealed that the difference between TyG-WHtR and TyG-WC did not reach statistical significance (*P* = 0.492; **Supplementary Table 3**), which indicates comparable overall discriminative ability. Given its numerically superior performance and the practical advantages of its components, TyG-WHtR was selected for further threshold analysis. The optimal cut-off value for the TyG-WHtR was determined to be 4.54 based on the Youden index, yielding a sensitivity of 63.1% and a specificity of 66.9%. Participants were subsequently stratified into low-risk (< 4.54) and high-risk (≥ 4.54) groups according to this threshold. Kaplan–Meier analysis revealed a significant difference in survival curves between the two groups (**Figure 3**), and the log-rank test confirmed a statistically significant difference (*P* < 0.001). Detailed diagnostic metrics (including sensitivity, specificity, likelihood ratios, and predictive values) for all 12 metabolic indices are summarized in **Supplementary Table 4**.

### Time-dependent predictive performance of TyG-WHtR

**Figure 4:**
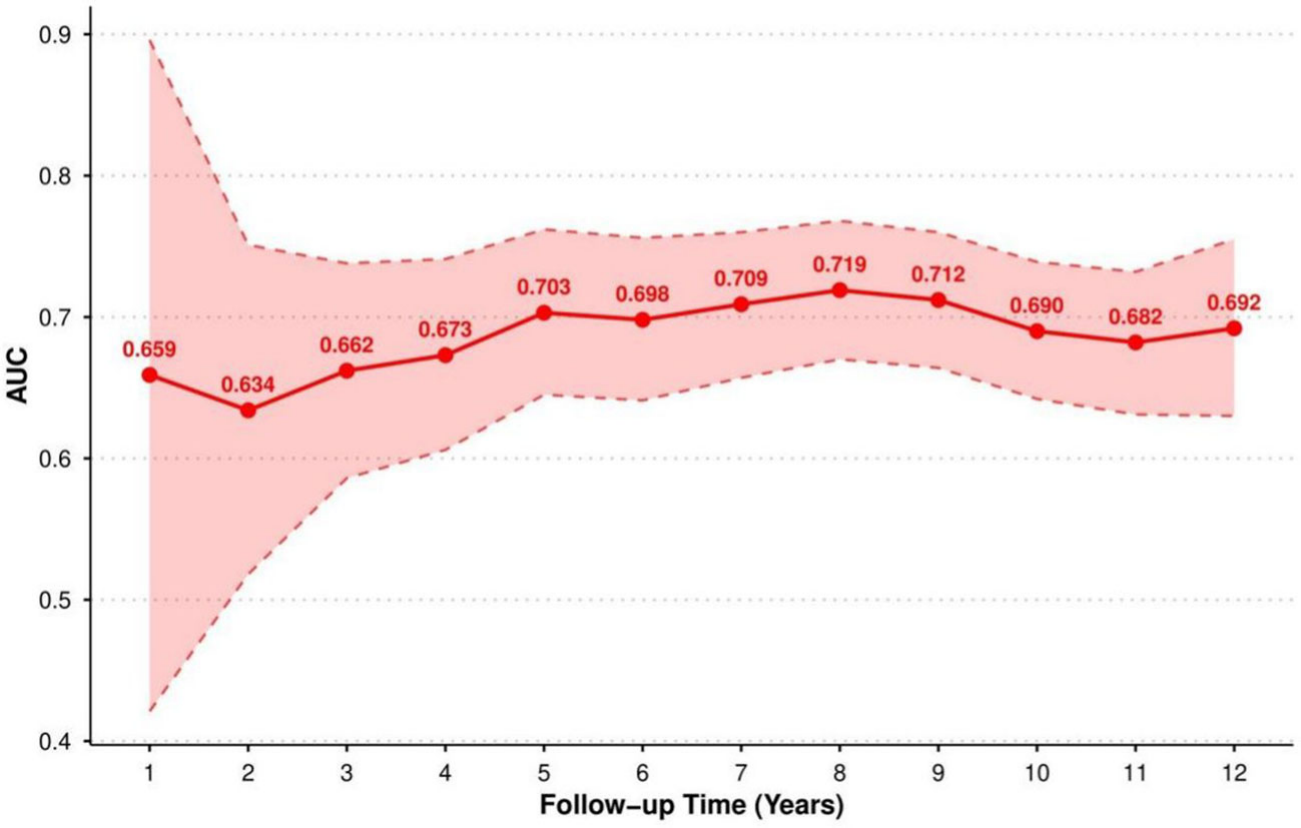
The ability of TyG-WHtR to predict T2DM incidence at time points from 1 to 12 years.

Time-dependent ROC analysis revealed the dynamic predictive performance of the TyG-WHtR during follow-up (**Figure 4**, **Supplementary Figure 3**). The predictive accuracy of this index improved with longer follow-up duration, as AUC values stabilized between 0.703 and 0.719 during the 5- to 10-year period. The highest predictive performance was observed at 8 years (AUC = 0.719, 95%CI: 0.670–0.768). These findings suggest that the TyG-WHtR is a reliable mid- to long-term predictor of T2DM in patients with NAFLD.

## Discussion

In this secondary analysis of a prospective Japanese cohort, we found that all twelve metabolic composite indices were significantly and linearly associated with incident T2DM in patients with NAFLD. Among these indices, the TyG-WHtR demonstrated superior predictive performance for T2DM onset, with a numerically higher AUC and stable long-term discriminatory ability. These findings suggest that the TyG-WHtR may serve as a practical and reliable tool for long-term T2DM risk stratification in the NAFLD population, which will facilitate early identification and timely clinical intervention in this high-risk group.

Our findings are consistent with a growing body of evidence that has established metabolic composite indices as effective predictors of T2DM. Among the various indices, TyG-WHtR has been identified as a particularly promising predictor; for instance, Dou et al. reported that this index exhibited the highest hazard ratio (HR = 2.99, 95% CI: 2.45–3.65) among sixteen glycolipid indicators in the general population (20). Other TyG-related indices have also demonstrated reliable associations. Using data from the China Health and Retirement Longitudinal Study (CHARLS), Li et al. reported that the TyG index was associated with T2DM risk in adults aged ≥45 years (HR = 1.75; 95% CI: 1.56–1.97) (21). Similarly, TyG-BMI and TyG-WC have been validated as predictors of T2DM (22), and a recent study confirmed the predictive value of the triglyceride-glucose-weight-adjusted waist index (TyG-WWI) (23).

Beyond TyG-based metrics, indices that reflect insulin resistance and lipid accumulation have demonstrated consistent predictive ability. In a cohort of 7,583 middle-aged and older Chinese adults, Cheng et al. reported that each increase in the METS-IR was associated with a 33% increased risk of incident T2DM (HR = 1.33, 95% CI: 1.22–1.45) (24). Furthermore, a large-scale study by Ma et al. involving 195,989 Japanese participants confirmed that the AIP, cardiometabolic index (CMI), LAP, and VAI were significantly associated with increased T2DM risk (25). Additional indices, such as the CHG (26, 27), have also been linked to T2DM incidence, which collectively underscores the utility of composite metabolic markers for diabetes risk assessment across diverse populations.

Previous studies have demonstrated that the TyG-BRI is associated with cardiovascular diseases (28), cardiometabolic multimorbidity (CMM) (29), stroke (30) and chronic kidney disease (CKD) (31). However, to the best of our knowledge, no study has investigated the association between the TyG-BRI and incident T2DM. Our findings indicate that among individuals with NAFLD, the TyG-BRI is linearly associated with the risk of developing new-onset T2DM.

In the present study, all twelve metabolic composite indices were linearly associated with T2DM risk in NAFLD patients. In contrast, Dou et al. reported nonlinear associations for several indices, including TyG-BMI, METS-IR, CMI, VAI, and LAP, in the general population (20). This discrepancy likely reflects differences in baseline metabolic risk; NAFLD patients, by virtue of their condition, may have already surpassed the threshold where nonlinear effects are observed, resulting in a dose–response pattern that is more uniform across the exposure spectrum.

Although the AUC values (0.68–0.72) suggest only moderate discrimination, the TyG-WHtR consistently outperforms the other assessed indices. This finding indicates that while it may not serve as a definitive standalone diagnostic tool, the TyG-WHtR offers superior risk stratification compared with other indices, which suggests that it may be a valuable predictor of incident T2DM. This observation is consistent with the findings of Dou et al., who also identified the TyG-WHtR as a promising predictor among multiple indicators (20). However, other studies have reported different useful predictors. For example, using data from the National Health and Nutrition Examination Survey (NHANES), Tu et al. reported that the triglyceride-glucose-weight-adjuested waist index (TyG-WWI) had the highest predictive accuracy (AUC = 0.81) (23) for T2DM, whereas Zhang et al. reported that the TyG index performed best in both males and females in the CHARLS cohort, (AUC = 0.78) (32). These variations likely reflect differences in study populations, including geographic, ethnic, age and lifestyle factors, which may influence body composition, lipid profiles, and, consequently, the performance of metabolic indices.

A key practical finding was the optimal TyG-WHtR cut-off of 4.54 for the prediction of T2DM in NAFLD patients. Individuals exceeding this threshold had a significantly higher cumulative incidence of T2DM (log-rank P<0.001). Notably, this value closely approximates the cut-off of 4.71 reported in a middle-aged Chinese cohort (32), suggesting that the risk threshold of TyG-WHtR may be relatively consistent across East Asian populations, even in the presence of NAFLD.

T2DM is a complex metabolic disorder whose pathogenesis is not fully understood, although IR is widely recognized as a core mechanism (33). The TyG index, a validated surrogate marker of IR (34–36), was also associated with T2DM risk in our NAFLD cohort. The predictive utility of TyG may stem from its components—FPG and TG. Elevated TG levels can exacerbate IR through pathways such as impaired insulin signaling, proinflammatory cytokine release, oxidative stress, and disruption of intracellular pathways (37). Elevated FPG is often indicative of hepatic IR (38, 39). Obesity, particularly central adiposity, is another established risk factor for T2DM (40–42). While BMI is typically used to assess overall obesity, it does not capture fat distribution. In contrast, WC and related indices, such as the waist–height ratio (WHtR), body roundness index (BRI), and weight-adjusted waist index (WWI), are more sensitive markers of abdominal fat accumulation. Among these, the WHtR has been suggested to be the strongest predictor of T2DM (43–45). The integration of TyG (reflecting IR) with WHtR (reflecting central obesity) may thus provide a more comprehensive risk assessment, which is supported by our findings.

Our findings should be interpreted in the context of the recent study by Dou et al., which analyzed similar indices in a general population. While Dou et al. reported that the TyG-WHtR was a robust and promising predictor of T2DM, our study extends this knowledge by demonstrating that the TyG-WHtR retains its stable predictive value even in the presence of hepatic steatosis. The absolute AUC values and the relative rankings of the 12 indices in our NAFLD cohort highlight the importance of population-specific validation. In addition, we identified and validated the optimal cut-off value for TyG-WHtR. These findings will help clinicians utilize this index to identify high-risk individuals among NAFLD patients.

Several strengths of our study deserve mention. First, we identified a readily accessible and clinically feasible indicator that may help health care professionals stratify NAFLD patients according to their T2DM risk. Second, our study compared a broad panel of twelve metabolic composite indices, providing a more comprehensive evaluation than previous reports. Third, the use of time-ROC analysis allowed us to assess the stability of the predictive ability of the TyG-WHtR across different time points.

Nevertheless, this study has several limitations. First, regarding external validity, our study cohort consisted exclusively of Japanese individuals. Given the known ethnic variations in body composition and fat distribution, particularly the evidence that Asians often develop T2DM at lower BMI levels than do Europeans (46), the generalizability of our findings to other ethnic or racial groups remains uncertain and requires validation in multiethnic cohorts. Second, the study population was predominantly male (~80%). Since sex differences in lipid metabolism, visceral adiposity, and NAFLD progression are well documented (47–49), our results, including the cut-off value, may not be fully applicable to female patients. Future studies involving more balanced sex ratios and diverse ethnic groups are warranted to confirm the universal applicability of the TyG-WHtR as a predictive tool. Third, the absence of oral glucose tolerance test (OGTT) data may have led to an underestimation of T2DM incidence and potential bias in risk assessment. Fourth, as a secondary analysis of the NAGALA cohort, unmeasured confounding factors from the original study could not be fully addressed.

In summary, all twelve metabolic composite indices were independently and linearly associated with incident T2DM in NAFLD patients. Among them, the TyG-WHtR emerged as a promising predictor, with an optimal cut-off of 4.54 for the stratification of high-risk individuals. These findings support the use of the TyG-WHtR as a practical indicator for early risk identification and targeted intervention in this high-risk population.

## Data Availability

The datasets presented in this study can be found in online repositories. The data are available at https://datadryad.org/dataset/doi:10.5061/dryad.8q0p192.
