# Supplementary figures and images for: TyG-WHtR predicts incident type 2 diabetes mellitus in NAFLD: a 12-year prospective cohort study
Source: Front Endocrinol (Lausanne). 2026 May 1;17:1805902. doi: 10.3389/fendo.2026.1805902 (PMC13175847; doi:10.3389/fendo.2026.1805902)

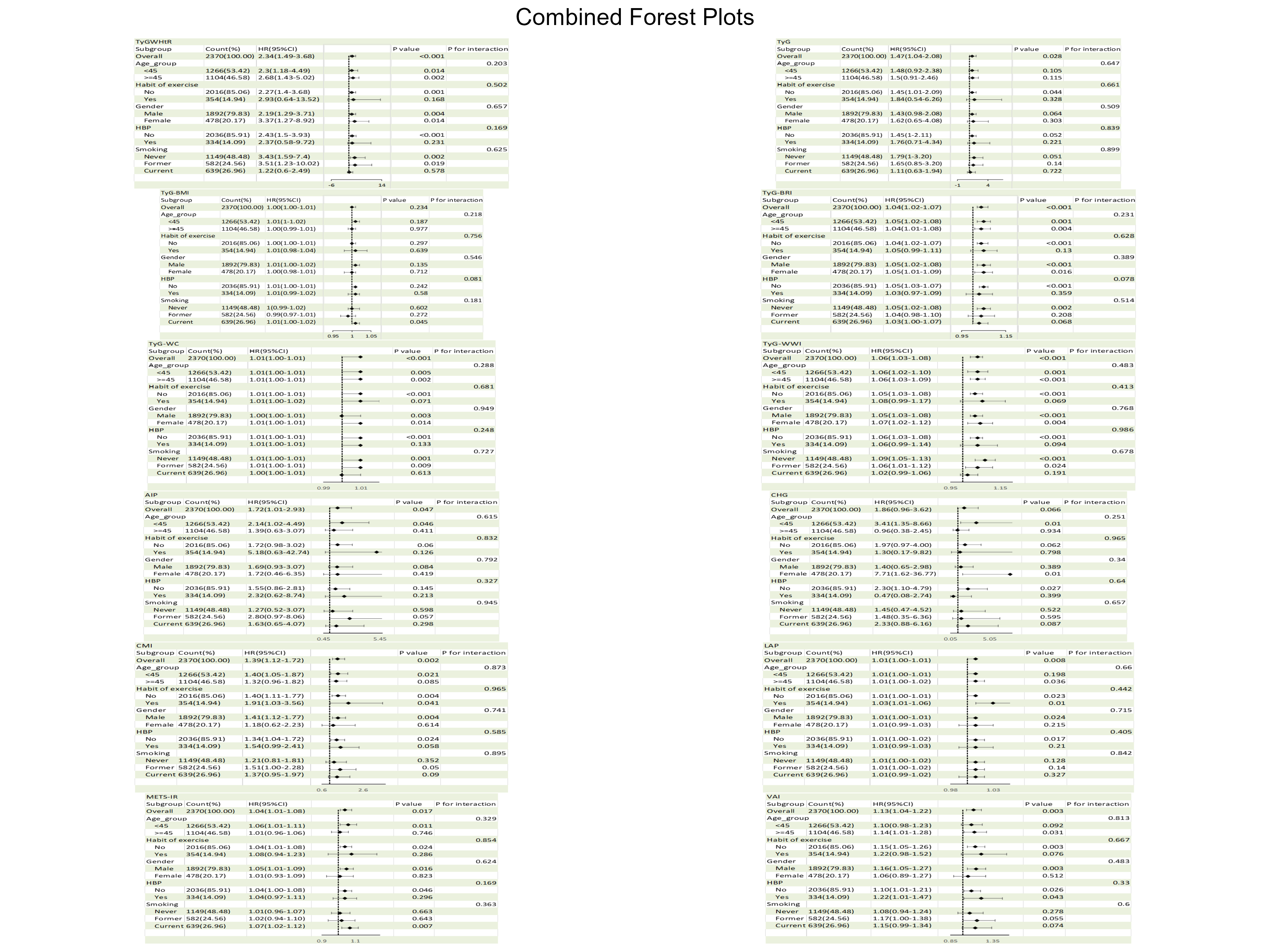

Supplement: Supplementary file 1 [file Image1.png]

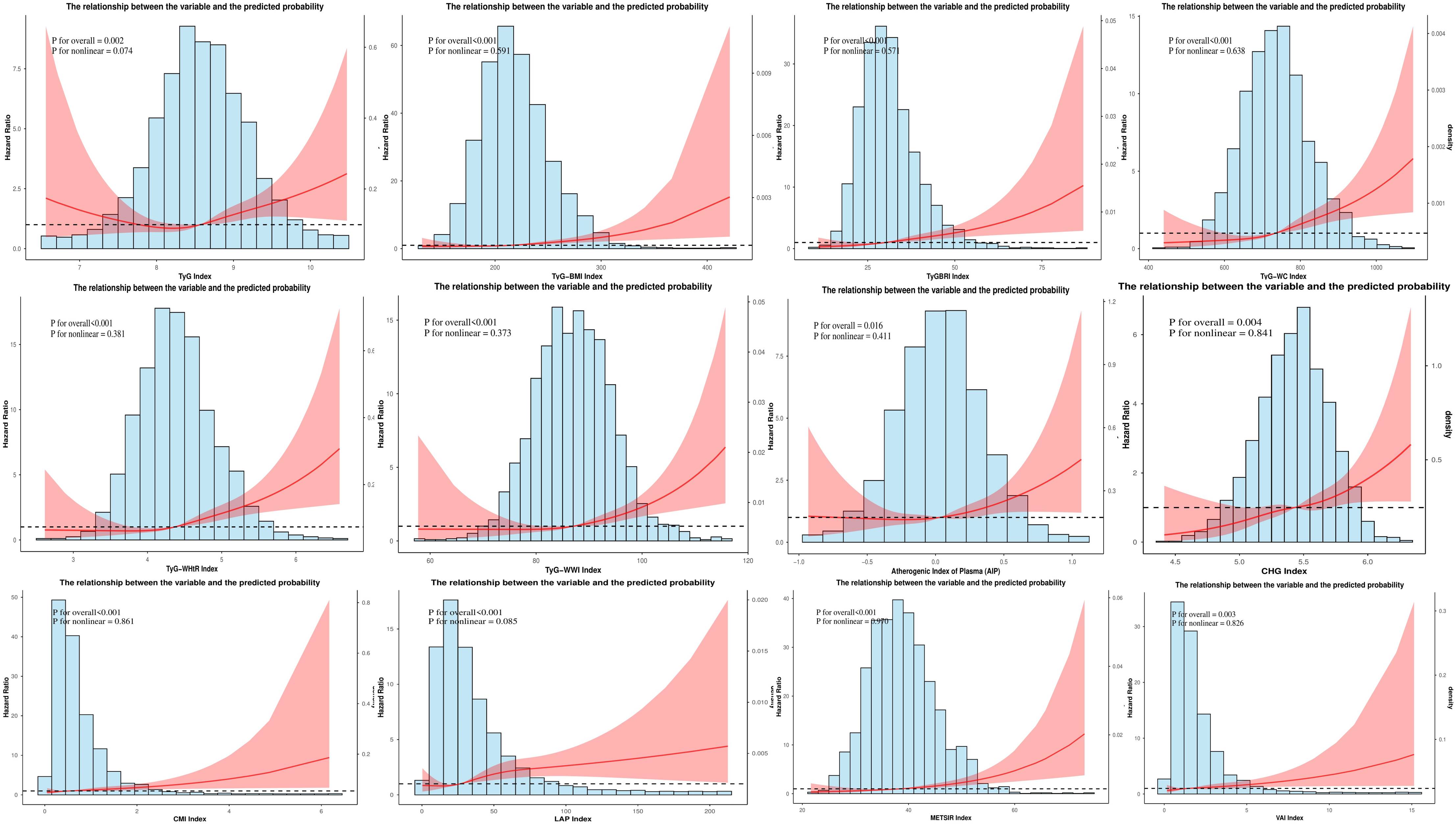

Supplement: Supplementary file 2 [file Image2.jpeg]

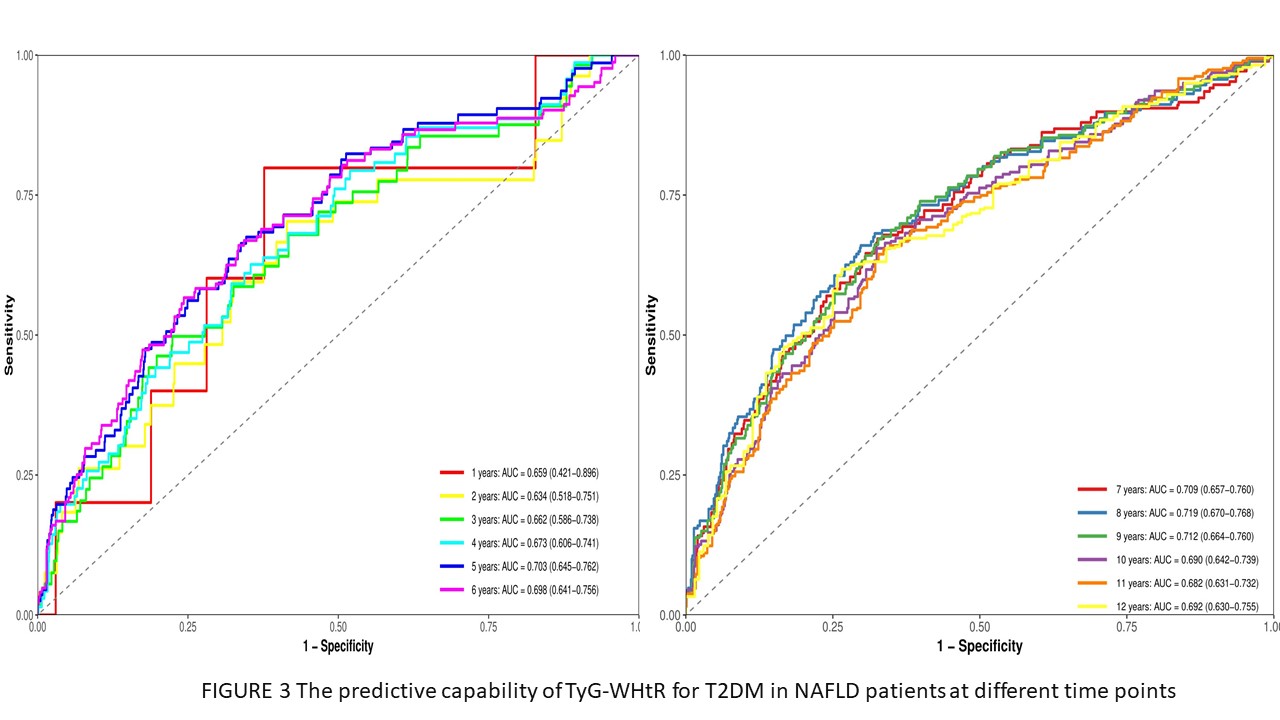

Supplement: Supplementary file 3 [file Image3.jpeg]
