# Supplementary material for: TyG-WHtR predicts incident type 2 diabetes mellitus in NAFLD: a 12-year prospective cohort study
Source: Front Endocrinol (Lausanne). 2026 May 1;17:1805902. doi: 10.3389/fendo.2026.1805902 (PMC13175847; doi:10.3389/fendo.2026.1805902)
Supplement: Supplementary file 4 [file Table1.docx]

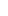
Supplementary Table1 The formulas of indices

| Categories | Definition |
| --- | --- |
| BMI | weight (kg) / height(m)^2^ |
| WHtR | WC (cm)/ height (cm) |
| WWI | WC(cm)/ weight(kg)^0.5^ |
| BRI | 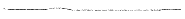364.2-365.5×√[1-(WC(cm)/(π×height(cm)))^2^] |
| TyG | Ln [(TG (mg/dL) × FPG(mg/dL)) /2] |
| TyG-BMI | TyG ×BMI |
| TyG-WC | TyG ×WC(cm) |
| TyG-WHtR | TyG ×WHtR |
| TyG-BRI | TyG ×BRI |
| TyG-WWI | TyG ×WWI |
| CHG | Ln [TC (mg/dL)×FPG(mg/dL) /(2×HDL-c (mg/dL))] |
| METS-IR | [（Ln(2×FPG(mg/dL)+TG(mg/dL)) ×BMI(kg/m^2^)］/Ln（HDL-c(mg/dL)） |
| AIP | Lg[TG(mg/dL)/HDL-c(mg/dL)] |
| VAI | Males: WC (cm)/(39.68 + 1.88×BMI(kg/m^2^)) × TG(mmol/L)/1.03 ×1.31/HDL-c (mmol/L)  Females: WC (cm)/(36.58 + 1.89×BMI(kg/m^2^)) × TG(mmol/L)/0.81 ×1.52/HDL-c(mmol/L) |
| LAP | Males: (WC(cm) – 65) × TG(mmol/L)  Females: (WC(cm) – 58) × TG(mmol/L) |
| CMI | TG(mmol/L)/HDL-c(mmol/L) × WHtR |

BMI, body mass index; WHtR, waist-to-height ratio; WC, Waist circumference; AIP, atherogenic index of plasma; BRI, body roundness index; CHG, cholesterol, high density lipoprotein, and glucose index; CMI, cardiometabolic index; LAP, lipid accumulation product; METS-IR, metabolic score for insulin resistance; TyG, triglyceride-glucose index; WWI, weight-adjusted-waist index; VAI, visceral adiposity index; FPG, fasting plasma glucose


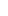

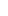
​​​
