# Supplementary material for: TyG-WHtR predicts incident type 2 diabetes mellitus in NAFLD: a 12-year prospective cohort study
Source: Front Endocrinol (Lausanne). 2026 May 1;17:1805902. doi: 10.3389/fendo.2026.1805902 (PMC13175847; doi:10.3389/fendo.2026.1805902)
Supplement: Supplementary file 5 [file Table2.docx]

Supplementary Table 2A Proportional hazards assumption testing using Schoenfeld residuals for all the variables in Model2

| TyG | | | TyG-BRI | | | TyG-BMI | | | TyG-WC | | |
| --- | --- | --- | --- | --- | --- | --- | --- | --- | --- | --- | --- |
| Variable | χ2 | P value | Variable | χ2 | P value | Variable | χ2 | P value | Variable | χ2 | P value |
| TyG | 0.13 | 0.72 | TyG-BRI | 0.25 | 0.62 | TyG-BMI | 1.18 | 0.28 | TyG-WC | 1.30 | 0.25 |
| Age | 0.62 | 0.43 | Age | 0.45 | 0.50 | Age | 0.54 | 0.46 | Age | 0.48 | 0.49 |
| ALT | 0.00 | 0.95 | ALT | 0.22 | 0.64 | ALT | 0.08 | 0.78 | ALT | 0.16 | 0.69 |
| AST | 0.34 | 0.56 | AST | 0.95 | 0.33 | AST | 0.72 | 0.40 | AST | 0.86 | 0.35 |
| GGT | 0.85 | 0.36 | GGT | 0.96 | 0.33 | GGT | 0.87 | 0.35 | GGT | 1.39 | 0.24 |
| HE | 0.24 | 0.62 | HE | 0.22 | 0.64 | HE | 0.26 | 0.61 | HE | 0.19 | 0.66 |
| HBP | 1.52 | 0.22 | HBP | 1.80 | 0.18 | HBP | 1.95 | 0.16 | HBP | 1.99 | 0.16 |
| Smoking | 2.25 | 0.33 | Smoking | 2.11 | 0.35 | Smoking | 2.14 | 0.34 | Smoking | 1.93 | 0.38 |
| gender | 0.85 | 0.36 | gender | 1.94 | 0.16 | gender | 0.87 | 0.35 | gender | 1.86 | 0.17 |

TABLE 2A (continued)

| TyG-WHtR | | | TyG-WWI | | | AIP | | | CHG | | |
| --- | --- | --- | --- | --- | --- | --- | --- | --- | --- | --- | --- |
| Variable | χ2 | P value | Variable | χ^2^ | P value | Variable | χ2 | P value | Variable | χ2 | P value |
| TyG-WHtR | 0.42 | 0.51 | TyG-WWI | 0.00 | 0.99 | AIP | 0.30 | 0.58 | CHG | 4.46 | 0.05 |
| Age | 0.60 | 0.44 | Age | 0.67 | 0.41 | Age | 0.60 | 0.44 | Age | 0.64 | 0.42 |
| ALT | 0.18 | 0.67 | ALT | 0.09 | 0.77 | ALT | 0.00 | 0.98 | ALT | 0.00 | 0.96 |
| AST | 0.86 | 0.35 | AST | 0.56 | 0.45 | AST | 0.32 | 0.57 | AST | 0.29 | 0.59 |
| GGT | 1.26 | 0.26 | GGT | 1.25 | 0.26 | GGT | 0.69 | 0.41 | GGT | 0.80 | 0.37 |
| HE | 0.18 | 0.67 | HE | 0.18 | 0.67 | HE | 0.28 | 0.60 | HE | 0.37 | 0.54 |
| HBP | 1.86 | 0.17 | HBP | 1.63 | 0.20 | HBP | 1.34 | 0.25 | HBP | 1.29 | 0.26 |
| Smoking | 2.03 | 0.36 | Smoking | 2.15 | 0.34 | Smoking | 2.29 | 0.32 | Smoking | 2.19 | 0.33 |
| gender | 2.06 | 0.15 | gender | 2.13 | 0.14 | gender | 0.66 | 0.42 | gender | 0.86 | 0.35 |

TABLE 2A (continued)

| CMI | | | | LAP | | | | METS-IR | | | | VAI | | |
| --- | --- | --- | --- | --- | --- | --- | --- | --- | --- | --- | --- | --- | --- | --- |
| Variable | χ2 | P value | Variable | | χ2 | P value | Variable | | χ2 | P value | Variable | | χ2 | P value |
| CMI | 0.02 | 0.89 | LAP | | 0.00 | 0.96 | METS-IR | | 1.96 | 0.16 | VAI | | 0.02 | 0.88 |
| Age | 0.49 | 0.48 | Age | | 0.47 | 0.49 | Age | | 0.60 | 0.44 | Age | | 0.49 | 0.48 |
| ALT | 0.00 | 0.95 | ALT | | 0.04 | 0.85 | ALT | | 0.06 | 0.81 | ALT | | 0.00 | 0.95 |
| AST | 0.36 | 0.55 | AST | | 0.55 | 0.46 | AST | | 0.64 | 0.42 | AST | | 0.35 | 0.55 |
| GGT | 0.85 | 0.36 | GGT | | 1.49 | 0.22 | GGT | | 0.87 | 0.35 | GGT | | 0.82 | 0.36 |
| HE | 0.20 | 0.66 | HE | | 0.21 | 0.64 | HE | | 0.23 | 0.63 | HE | | 0.21 | 0.65 |
| HBP | 1.45 | 0.23 | HBP | | 2.00 | 0.16 | HBP | | 1.51 | 0.22 | HBP | | 1.36 | 0.24 |
| Smoking | 2.18 | 0.34 | Smoking | | 2.05 | 0.36 | Smoking | | 2.03 | 0.36 | Smoking | | 2.26 | 0.32 |
| gender | 0.66 | 0.42 | gender | | 1.07 | 0.30 | gender | | 0.70 | 0.40 | gender | | 0.69 | 0.41 |

ALT, alanine aminotransferase; AST, aspartate transaminase; GGT, gamma-glutamyl transferase; BMI, body mass index; WC, Waist circumference; WHtR, waist-to-height ratio; AIP, atherogenic index of plasma; BRI, body roundness index; CHG, cholesterol, high density lipoprotein, and glucose index; CMI, cardiometabolic index; LAP, lipid accumulation product; METS-IR, metabolic score for insulin resistance; TyG, triglyceride-glucose index; WWI, weight-adjusted-waist index; VAI, visceral adiposity index; HBP, hypertension;
