# Supplementary material for: TyG-WHtR predicts incident type 2 diabetes mellitus in NAFLD: a 12-year prospective cohort study
Source: Front Endocrinol (Lausanne). 2026 May 1;17:1805902. doi: 10.3389/fendo.2026.1805902 (PMC13175847; doi:10.3389/fendo.2026.1805902)
Supplement: Supplementary file 6 [file Table3.docx]

Supplementary Table2B the VIF of all the variables in Model2

| TyG | | TyG-BRI | | TyG-BMI | | TyG-WC | |
| --- | --- | --- | --- | --- | --- | --- | --- |
| Variable | VIF | Variable | VIF | Variable | VIF | Variable | VIF |
| gender | 1.360 | gender | 1.399 | gender | 1.344 | gender | 1.363 |
| Age | 1.130 | Age | 1.130 | Age | 1.150 | Age | 1.128 |
| ALT | 3.693 | ALT | 3.734 | ALT | 3.734 | ALT | 3.730 |
| AST | 3.259 | AST | 3.253 | AST | 3.253 | AST | 3.253 |
| HE | 1.017 | HE | 1.020 | HE | 1.017 | HE | 1.020 |
| GGT | 1.315 | GGT | 1.283 | GGT | 1.287 | GGT | 1.291 |
| Smoking | 1.193 | Smoking | 1.191 | Smoking | 1.195 | Smoking | 1.196 |
| HBP | 1.033 | HBP | 1.070 | HBP | 1.085 | HBP | 1.066 |
| TyG | 1.128 | TyGBRI | 1.183 | TyGBMI | 1.214 | TyGWC | 1.228 |

TABLE 2B (continued)

| TyG-WHtR | | TyG-WWI | | AIP | | CHG | |
| --- | --- | --- | --- | --- | --- | --- | --- |
| Variable | VIF | Variable | VIF | Variable | VIF | Variable | VIF |
| gender | 1.362 | gender | 1.353 | gender | 1.374 | gender | 1.398 |
| Age | 1.132 | Age | 1.184 | Age | 1.129 | Age | 1.136 |
| ALT | 3.743 | ALT | 3.717 | ALT | 3.700 | ALT | 3.728 |
| AST | 3.254 | AST | 3.257 | AST | 3.261 | AST | 3.268 |
| HE | 1.020 | HE | 1.022 | HE | 1.018 | HE | 1.021 |
| GGT | 1.293 | GGT | 1.304 | GGT | 1.295 | GGT | 1.284 |
| Smoking | 1.195 | Smoking | 1.192 | Smoking | 1.203 | Smoking | 1.208 |
| HBP | 1.066 | HBP | 1.037 | HBP | 1.032 | HBP | 1.030 |
| TyGWHtR | 1.178 | TyGWWI | 1.155 | AIP | 1.137 | CHG | 1.196 |

TABLE 2B (continued)

| CMI | | LAP | | METS-IR | | VAI | |
| --- | --- | --- | --- | --- | --- | --- | --- |
| Variable | VIF | Variable | VIF | Variable | VIF | Variable | VIF |
| gender | 1.350 | gender | 1.345 | gender | 1.343 | gender | 1.346 |
| Age | 1.128 | Age | 1.128 | Age | 1.149 | Age | 1.128 |
| ALT | 3.688 | ALT | 3.699 | ALT | 3.738 | ALT | 3.683 |
| AST | 3.254 | AST | 3.253 | AST | 3.253 | AST | 3.255 |
| HE | 1.018 | HE | 1.019 | HE | 1.018 | HE | 1.018 |
| GGT | 1.293 | GGT | 1.302 | GGT | 1.280 | GGT | 1.293 |
| Smoking | 1.201 | Smoking | 1.197 | Smoking | 1.204 | Smoking | 1.199 |
| HBP | 1.035 | HBP | 1.053 | HBP | 1.076 | HBP | 1.032 |
| CMI | 1.092 | LAP | 1.120 | METSIR | 1.204 | VAI | 1.046 |

ALT, alanine aminotransferase; AST, aspartate transaminase; GGT, gamma-glutamyl transferase; BMI, body mass index; WC, Waist circumference; WHtR, waist-to-height ratio; AIP, atherogenic index of plasma; BRI, body roundness index; CHG, cholesterol, high density lipoprotein, and glucose index; CMI, cardiometabolic index; LAP, lipid accumulation product; METS-IR, metabolic score for insulin resistance; TyG, triglyceride-glucose index; WWI, weight-adjusted-waist index; VAI, visceral adiposity index; HBP, hypertension; VIF, variance inflation factor
