# Supplementary material for: TyG-WHtR predicts incident type 2 diabetes mellitus in NAFLD: a 12-year prospective cohort study
Source: Front Endocrinol (Lausanne). 2026 May 1;17:1805902. doi: 10.3389/fendo.2026.1805902 (PMC13175847; doi:10.3389/fendo.2026.1805902)
Supplement: Supplementary file 7 [file Table4.docx]

Supplementary Table 2C Multivariable Cox proportional hazards regression analysis（sensitive analysis ）

| Variables | Unadjusted model | | Model 1 | | Model 2 | |
| --- | --- | --- | --- | --- | --- | --- |
|  | HR (95%CI) | *P* value | HR (95%CI) | *P* value | HR (95%CI) | *P* value |
| **TyG** |  |  |  |  |  |  |
| ^1^Continuous | 2.03(1.57-2.63) | <0.001 | 2.21(1.69-2.88) | <0.001 | 1.60(1.21-2.11) | <0.001 |
| ^2^Continuous | 1.48(1.28-1.70) | <0.001 | 1.54(1.33-1.79) | <0.001 | 1.29(1.10-1.50) | <0.001 |
| Categorized |  |  |  |  |  |  |
| Low [6.56,8.34) | Reference |  | Reference |  | Reference |  |
| Middle [8.34,8.81) | 1.31(0.87-1.97) | 0.20 | 1.36(0.90-2.05) | 0.14 | 1.07(0.71-1.62) | 0.74 |
| High [8.81-10.47] | 2.46(1.71-3.54) | <0.001 | 2.71(1.87-3.95) | <0.001 | 1.73(1.18-2.54) | 0.005 |
| P for trend |  | <0.001 |  | <0.001 |  | 0.002 |
| **TyG-BRI** |  |  |  |  |  |  |
| ^1^Continuous | 1.06(1.05-1.08) | <0.001 | 1.07(1.05-1.08) | <0.001 | 1.05(1.04-1.07) | <0.001 |
| ^2^Continuous | 1.68(1.51-1.87) | <0.001 | 1.71(1.53-1.91) | <0.001 | 1.51(1.33-1.71) | <0.001 |
| Categorized |  |  |  |  |  |  |
| Low [10.84,27.12) | Reference |  | Reference |  | Reference |  |
| Middle [27.12,33.29) | 1.94(1.26-3.00) | 0.003 | 1.86(1.20-2.88) | 0.005 | 1.95(1.26-3.03) | 0.003 |
| High [33.29,86.87] | 3.77(2.52-5.63) | <0.001 | 3.62(2.42-5.42) | <0.001 | 2.80(1.86-4.23) | <0.001 |
| P for trend |  | <0.001 |  | <0.001 |  | <0.001 |
| **TyG-BMI** |  |  |  |  |  |  |
| ^1^Continuous | 1.02(1.01-1.02) | <0.001 | 1.02(1.01-1.02) | <0.001 | 1.01(1.01-1.02) | <0.001 |
| ^2^Continuous | 1.76(1.56-2.02) | <0.001 | 1.91(1.67-2.18) | <0.001 | 1.62(1.40-1.86) | <0.001 |
| Categorized |  |  |  |  |  |  |
| Low [131.17,203.68) | Reference |  | Reference |  | Reference |  |
| Middle[203.68-230.14) | 1.59(1.05-2.42) | 0.03 | 1.65(1.08-2.52) | 0.02 | 1.53(1.00-2.34) | 0.05 |
| High [230.14,421.35] | 3.31(2.26-4.86) | <0.001 | 3.76(2.55-5.53) |  | 2.50(1.67-3.74) | <0.001 |
| P for trend |  | <0.001 |  | <0.001 |  | <0.001 |
| **TyG-WC** |  |  |  |  |  |  |
| ^2^Continuous | 1.76(1.56-2.02) | <0.001 | 1.91(1.67-2.18) | <0.001 | 1.62(1.40-1.86) | <0.001 |
| ^1^Continuous | 1.01(1.01-1.01) | <0.001 | 1.01(1.01-1.01) | <0.001 | 1.01(1.00-1.01) | <0.001 |
| Categorized |  |  |  |  |  |  |
| Low [440.11,697.66) | Reference |  | Reference |  | Reference |  |
| Middle [697.66,770.64) | 1.39(0.90-2.15) | 0.11 | 1.64(1.04-2.57) | 0.03 | 1.38(0.88-2.17) | 0.17 |
| High [770.64,1097.18] | 3.36(2.29-4.93) | <0.001 | 4.12(2.75-6.17) | <0.001 | 2.82(1.86-4.28) | <0.001 |
| P for trend |  | <0.001 |  | <0.001 |  | <0.001 |
| **TyG-WHtR** |  |  |  |  |  |  |
| ^1^Continuous | 3.43(2.66-4.41) | <0.001 | 3.45(2.67-4.46) | <0.001 | 2.51(1.91-3.31) | <0.001 |
| ^2^Continuous | 1.88(1.65-2.14) | <0.001 | 1.88(1.65-2.14) | <0.001 | 1.59(1.39-1.83) | <0.001 |
| Categorized |  |  |  |  |  |  |
| Low [2.62,4.17) | Reference |  | Reference |  | Reference |  |
| Middle [4.17,4.58) | 1.44(0.92-2.25) | 0.11 | 1.42(0.91-2.23) | 0.12 | 1.22(0.78-1.91) | 0.39 |
| High [4.58,6.59] | 3.97(2.68-5.87) | <0.001 | 3.95(2.67-5.84) | <0.001 | 2.64(1.76-3.95) | <0.001 |
| P for trend |  | <0.001 |  | <0.001 |  | <0.001 |

TABLE 2C (continued)

| Variables | Unadjusted model | | Model 1 | | Model 2 | |
| --- | --- | --- | --- | --- | --- | --- |
|  | HR (95%CI) | *P* value | HR (95%CI) | *P* value | HR (95%CI) | *P* value |
| **TyG-WWI** |  |  |  |  |  |  |
| ^1^Continuous | 1.09(1.07-1.11) | <0.001 | 1.08(1.06-1.10) | <0.001 | 1.06(1.04-1.08) | <0.001 |
| ^2^Continuous | 1.83(1.59-2.11) | <0.001 | 1.78(1.55-2.06) | <0.001 | 1.48(1.28-1.71) | <0.001 |
| Categorized |  |  |  |  |  |  |
| Low [57.74,83.77) | Reference |  | Reference |  | Reference |  |
| Middle [83.77,90.20) | 1.51(0.99-2.31) | 0.05 | 1.51(0.98-2.31) | 0.06 | 1.245(0.81-1.91) | 0.32 |
| High [90.20,115.75] | 3.11(2.13-4.56) | <0.001 | 3.00(2.04-4.43) | <0.001 | 2.11(1.43-3.12) | <0.001 |
| P for trend |  | <0.001 |  | <0.001 |  | <0.001 |
| **AIP** |  |  |  |  |  |  |
| ^1^Continuous | 2.88(1.77-4.70) | <0.001 | 3.46(2.09-5.72) | <0.001 | 2.16(1.28-3.62) | 0.004 |
| ^2^Continuous | 1.37(1.18-1.59) | <0.001 | 1.44(1.24-1.67) | <0.001 | 1.25(1.07-1.45) | <0.001 |
| Categorized |  |  |  |  |  |  |
| Low [-0.93, -0.097) | Reference |  | Reference |  | Reference |  |
| Middle [-0.097,0.15) | 1.42(0.96-2.11) | 0.08 | 1.52(1.02-2.26) | 0.04 | 1.54(1.03-2.29) | 0.04 |
| High [0.15,1.07] | 2.06(1.43-2.97) | <0.001 | 2.33(1.60-3.41) | <0.001 | 1.72(1.17-2.53) | 0.006 |
| P for trend |  | <0.001 |  | <0.001 |  | 0.007 |
| **CHG** |  |  |  |  |  |  |
| ^1^Continuous | 5.83(3.44-9.89) | <0.001 | 7.00(4.07-12.04) | <0.001 | 2.96(1.68-5.21) | <0.001 |
| ^2^Continuous | 1.64(1.41-1.90) | <0.001 | 1.72(1.48-2.00) | <0.001 | 1.34(1.15-1.57) | <0.001 |
| Categorized |  |  |  |  |  |  |
| Low [4.41,5.31) | Reference |  | Reference |  | Reference |  |
| Middle [5.31,5.54) | 2.12(1.38-3.26) | <0.001 | 2.29(1.48-3.54) | <0.001 | 1.98(1.28-3.06) | 0.002 |
| High [5.54,6.34] | 2.94(1.95-4.42) | <0.001 | 3.32(2.17-5.06) | <0.001 | 2.03(1.32-3.14) | 0.001 |
| P for trend |  | <0.001 |  | <0.001 |  | 0.003 |
| **CMI** |  |  |  |  |  |  |
| ^1^Continuous | 1.73(1.45-2.08) | <0.001 | 1.80(1.50-2.16) | <0.001 | 1.57(1.27-1.93) | <0.001 |
| ^2^Continuous | 1.35(1.22-1.48) | <0.001 | 1.37(1.25-1.51) | <0.001 | 1.27(1.13-1.42) | <0.001 |
| Categorized |  |  |  |  |  |  |
| Low [0.053,0.41) | Reference |  | Reference |  | Reference |  |
| Middle [0.41,0.74) | 1.33(0.89-2.00) | 0.16 | 1.43(0.96-2.15) | 0.08 | 1.29(0.85-1.94) | 0.23 |
| High [0.74,6.17] | 2.22(1.54-3.20) | <0.001 | 2.49(1.71-3.64) | <0.001 | 1.77(1.20-2.60) | 0.004 |
| P for trend |  | <0.001 |  | <0.001 |  | 0.002 |
| **LAP** |  |  |  |  |  |  |
| ^2^Continuous | 1.49(1.35-1.64) | <0.001 | 1.52(1.38-1.68) | <0.001 | 1.36(1.22-1.52) | <0.001 |
| ^1^Continuous | 1.02(1.01-1.02) | <0.001 | 1.02(1.01-1.02) | <0.001 | 1.01(1.01-1.02) | <0.001 |
| Categorized |  |  |  |  |  |  |
| Low [0.34,19.72) | Reference |  | Reference |  | Reference |  |
| Middle [19.72,35.54) | 1.42(0.92-2.20) | 0.11 | 1.42(0.92-2.19) | 0.12 | 1.12(0.72-1.74) | 0.61 |
| High [35.54,212.52] | 3.32(2.26-4.87) | <0.001 | 3.51(2.39-5.17) | <0.001 | 2.46(1.65-3.66) | <0.001 |
| P for trend |  | <0.001 |  | <0.001 |  | <0.001 |

TABLE 2C (continued)

| Variables | Unadjusted model | | Model 1 | | Model 2 | |
| --- | --- | --- | --- | --- | --- | --- |
|  | HR (95%CI) | *P* value | HR (95%CI) | *P* value | HR (95%CI) | *P* value |
| **METS-IR** |  |  |  |  |  |  |
| ^1^Continuous | 1.08(1.06-1.10) | <0.001 | 1.10(1.07-1.12) | <0.001 | 1.07(1.05-1.10) | <0.001 |
| ^2^Continuous | 1.63(1.44-1.84) | <0.001 | 1.78(1.56-2.02) | <0.001 | 1.55(1.34-1.78) | <0.001 |
| Categorized |  |  |  |  |  |  |
| Low [21.61,35.87) | Reference |  | Reference |  | Reference |  |
| Middle [35.87,41.08) | 1.16(0.77-1.76) | 0.48 | 1.25(0.83-1.91) | 0.29 | 1.13(0.74-1.73) | 0.57 |
| High [41.08,73.16] | 2.70(1.87-3.88) | <0.001 | 3.10(2.14-4.50) | <0.001 | 2.089(1.42-3.07) | <0.001 |
| P for trend |  | <0.001 |  | <0.001 |  | <0.001 |
| **VAI** |  |  |  |  |  |  |
| ^1^Continuous | 1.23(1.14-1.32) | <0.001 | 1.23(1.14-1.32) | <0.001 | 1.17(1.08-1.27) | <0.001 |
| ^2^Continuous | 1.32(1.20-1.45) | <0.001 | 1.32(1.20-1.49) | <0.001 | 1.24(1.11-1.38) | <0.001 |
| Categorized |  |  |  |  |  |  |
| Low [0.17,1.11) | Reference |  | Reference |  | Reference |  |
| Middle [1.11,1.92) | 1.40(0.94-2.09) | 0.10 | 1.44(0.96-2.15) | 0.08 | 1.32(0.88-1.98) | 0.18 |
| High [1.92,15.14] | 2.21(1.53-3.19) | <0.001 | 2.24(1.55-3.24) | <0.001 | 1.68(1.16-2.44) | 0.006 |
| P for trend |  | <0.001 |  | <0.001 |  | 0.006 |

Model1 adjust for sex, age,

Model2 adjust for sex age, ALT, AST, Habit of exercise, GGT, HbA1c, Smoking status, HBP

^1^HR per 1-unit increase

^2^HR per 1-SD increase

ALT, alanine aminotransferase; AST, aspartate transaminase; GGT, gamma-glutamyl transferase; HbA1-c, hemoglobin a1c; BMI, body mass index; WC, Waist circumference; WHtR, waist-to-height ratio; AIP, atherogenic index of plasma; BRI, body roundness index; CHG, cholesterol, high density lipoprotein, and glucose index; CMI, cardiometabolic index; LAP, lipid accumulation product; METS-IR, metabolic score for insulin resistance; TyG, triglyceride-glucose index; WWI, weight-adjusted-waist index; VAI, visceral adiposity index; HBP, hypertension
