# Supplementary material for: TyG-WHtR predicts incident type 2 diabetes mellitus in NAFLD: a 12-year prospective cohort study
Source: Front Endocrinol (Lausanne). 2026 May 1;17:1805902. doi: 10.3389/fendo.2026.1805902 (PMC13175847; doi:10.3389/fendo.2026.1805902)
Supplement: Supplementary file 8 [file Table5.docx]

Supplementary Table3 The results of DeLong’s test for TyG-WHtR, TyG-WC, TyGWWI, TyG-BRI and LAP

| Indicators | Z | p-value |
| --- | --- | --- |
| TyGWHtR - TyGWC | 0.687 | 0.492 |
| TyGWHtR - TyGWWI | -0.964 | 0.335 |
| TyGWHtR - TyGBRI | -1.644 | 0.100 |
| TyGWHtR - LAP | -2.270 | 0.023 |

BMI, body mass index; WC, Waist circumference; WHtR, waist-to-height ratio; LAP, lipid accumulation product; TyG, triglyceride-glucose index; WWI, weight-adjusted-waist index;
