# Supplementary material for: TyG-WHtR predicts incident type 2 diabetes mellitus in NAFLD: a 12-year prospective cohort study
Source: Front Endocrinol (Lausanne). 2026 May 1;17:1805902. doi: 10.3389/fendo.2026.1805902 (PMC13175847; doi:10.3389/fendo.2026.1805902)
Supplement: Supplementary file 9 [file Table6.docx]

Supplementary TABLE 4 Cut-off between AUC, sensitivity, specificity, LRs, and PVs for twelve metabolic indices to detect T2DM in NAFLD patients.

| N=2370 | TyG | TyG-BRI | TyG-BMI | TyG-WC | TyG-WHtR | TyG-WWI | AIP | CHG | CMI | LAP | METS-IR | VAI |
| --- | --- | --- | --- | --- | --- | --- | --- | --- | --- | --- | --- | --- |
| AUC | 0.626 | 0.667 | 0.647 | 0.675 | 0.680 | 0.670 | 0.610 | 0.653 | 0.627 | 0.660 | 0.651 | 0.627 |
| Optimal cutoff | 8.78 | 31.91 | 213.99 | 772.70 | 4.54 | 88.47 | 0.13 | 5.61 | 0.77 | 36.32 | 38.79 | 1.82 |
| Youden’s Index | 0.22 | 0.26 | 0.24 | 0.281 | 0.300 | 0.260 | 0.173 | 0.227 | 0.201 | 0.268 | 0.239 | 0.198 |
| Sensitivity | 0.566 | 0.636 | 0.742 | 0.581 | 0.631 | 0.657 | 0.520 | 0.460 | 0.5 | 0.566 | 0.692 | 0.540 |
| Specificity | 0.658 | 0.621 | 0.492 | 0.700 | 0.669 | 0.603 | 0.652 | 0.767 | 0.701 | 0.703 | 0.547 | 0.658 |
| PPV | 0.13 | 0.13 | 0.12 | 0.150 | 0.148 | 0.131 | 0.12 | 0.152 | 0.132 | 0.148 | 0.122 | 0.126 |
| NPV | 0.94 | 0.95 | 0.95 | 0.948 | 0.952 | 0.951 | 0.937 | 0.940 | 0.939 | 0.947 | 0.951 | 0.940 |
| (+) LR | 1.66 | 1.68 | 1.48 | 1.935 | 1.907 | 1.654 | 1.497 | 1.973 | 1.673 | 1.902 | 1.529 | 1.578 |
| (-) LR | 0.66 | 0.59 | 0.52 | 0.599 | 0.551 | 0.569 | 0.735 | 0.705 | 0.713 | 0.618 | 0.563 | 0.699 |

AUC, area under curve; PV, predictive value; PPV, positive predictive value; NPV, negative predictive value; LR, likelihood ratio; T2DM, type 2 diabetes mellitus; NAFLD, non-alcoholic fatty liver disease; LR, likelihood ratio; BMI, body mass index; WC, Waist circumference; WHtR, waist-to-height ratio; AIP, atherogenic index of plasma; BRI, body roundness index; CHG, cholesterol, high density lipoprotein, and glucose index; CMI, cardiometabolic index; LAP, lipid accumulation product; METS-IR, metabolic score for insulin resistance; TyG, triglyceride-glucose index; WWI, weight-adjusted-waist index; VAI, visceral fat index; HBP, hypertension
